# Coronavirus disease 2019 (COVID-19) manifestations during pregnancy in all three trimesters: A case series

**DOI:** 10.18502/ijrm.v19i2.8477

**Published:** 2021-02-21

**Authors:** Elham Askary, Tahereh Poordast, Zahra Shiravani, Mohammad Ashraf Ali, Atefeh Hashemi, Razieh Naseri, Shaghayegh Moradialamdarloo, Zinat Karimi, Elham Izanloo, Fatemeh Sadat Najib

**Affiliations:** ^1^Department of Obstetrics and Gynecology, School of Medicine, Infertility Research Center, Shiraz University of Medical Sciences, Shiraz, Iran.; ^2^Student Research Committee, Shiraz University of Medical Sciences, Shiraz, Iran.; ^3^Department of Obstetrics and Gynecology, School of Medicine, Shiraz University of Medical Sciences, Shiraz, Iran.

**Keywords:** Pregnancy, COVID-19, Maternal-fetal infection transmission, Vertical transmission of infectious disease, Papulosquamous skin diseases.

## Abstract

**Background:**

Coronavirus disease 2019 (COVID-19) pandemic has raised concerns about the susceptibility amongst different groups of the population. Pregnant women are one such group. This study was conducted to investigate the effect of COVID-19 on pregnancy and maternal/neonatal outcomes.

**Case presentation:**

This case series was conducted on 16 pregnant women with COVID-19 from March 21 to May 11, 2020. Clinical characteristics, pregnancy complications, medication used, maternal/neonatal outcomes, and fatality rate were investigated through this study. The mean age of the patients was 30.06 yrs. Patients from all three trimesters were included (1 in first, 5 in second, and 10 in the third trimesters). The most common clinical symptoms were shortness of breath (n = 10), dry cough (n = 10), myalgia (n = 8), and chills (n = 7). Also, three cases had papulosquamous skin lesions with fissuring. The most common laboratory results were leukocytosis (n = 8), increased liver enzymes (n = 6), elevated CRP (n = 5), and thrombocytopenia (n = 4). There was one case of maternal mortality, five of premature labor pain (PLP), two of preeclampsia, and two of placenta accreta. Twelve pregnancies were terminated (nine cesarean sections, three vaginal deliveries). Among neonates, we had 6 cases of preterm labor. All neonates had negative PCR results.

**Conclusion:**

Clinical manifestations and paraclinical results were similar to non-pregnant patients. There was no evidence of vertical transmission. PLP and premature rupture of membranes (PROM) were the most common complications in the second and third trimesters of pregnant COVID-19 women, which can lead to rupture of the uterus. Termination and delivery should be planned individually.

## 1. Introduction

Coronavirus disease 2019 (COVID-19) has caused a mass infection in 213 countries/territories thus far (1). On March 11, 2020, COVID-19 was declared as a pandemic by the World Health Organization (WHO) (2). The high number of infected cases has raised concerns about susceptible groups in the general population. Pregnant women constitute a concerning group.

Pregnancy causes physiologic changes in women's immune systems, which can have adverse effects on maternal and neonatal outcomes during viral infections (3). Previous studies shown a more severe course of the disease in pregnant women with influenza virus infection (4). Also, previous coronavirus epidemics, such as the severe acute respiratory syndrome (SARS) and the Middle-East respiratory syndrome (MERS), have shown to cause serious complications during pregnancy (5, 6).

As shown previously, SARS increased the rate of spontaneous miscarriage in the first trimester, preterm delivery, intrauterine growth restriction (IUGR), and maternal death in pregnant women. A case-controlled trial on SARS patients reported a more severe clinical course and poorer outcomes in pregnant women compared to non-pregnant patients (7-10). MERS has caused an increase in maternal death rate, premature delivery, and perinatal morbidity in pregnant women (6, 11).

Some studies have investigated the effect of COVID-19 during pregnancy. However, most of the studies were small case series from China investigating women during the third trimester of pregnancy (12-16). Therefore, more information is needed regarding the effect of COVID-19 during different trimesters of pregnancy to guide treatment and prevent complications in pregnant women with COVID-19.

In this study, we conducted a multicenter retrospective case series on pregnant women with COVID-19 to investigate the effect of the disease on pregnancy and maternal/neonatal outcomes. To the best of our knowledge, this is the largest case series from Iran to date.

## 2. Case presentation

### Study design and patient collection

In this case series, 16 pregnant women infected with COVID-19 who were referred to three university hospitals affiliated with Shiraz University of Medical Sciences from March 21 to May 11, 2020 were included. These pregnant women were identified by the discharge diagnosis. Pregnant women with confirmed for diagnosis of COVID-19 were included in this study. The confirmation was done if at least two of the following three criteria were met: (I) Positive reverse-transcriptase-polymerase-chain-reaction (RT-PCR) results for severe acute respiratory syndrome coronavirus 2 (*SARS*-*CoV*-*2*) (II) Abnormal imaging findings (chest CT scan or chest X-ray [CXR]) in favor of COVID-19; and (III) Clinical symptoms of viral pneumonia in favor of COVID-19.

### Data collection 

Data collection was done using paper medical records of the included patients and self-reported histories. Data, including patients' self-reported past medical histories, self-reported past medication histories, clinical characteristics, laboratory results, imaging findings, treatment course, and maternal/neonatal outcomes were collected.

### Ethical considerations

This study was approved by the ethics committee of Shiraz University of Medical Sciences (IR.SUMS.REC.1399.589) and in accordance with the Declaration of Helsinki. In addition, all patients gave a written informed consent for using their medical information in research projects.

### Results

The mean age of the patients was 30.06 years and ranged from 19 to 37 years old. One case was in the first, five in the second, and ten in the third trimester. Eight cases were hospitalized from the beginning with the impression of COVID-19, and the other cases were admitted due to other reasons such as preterm labor (n = 5), receiving chemotherapy for ovarian dysgerminoma in 20 wks of pregnancy (n = 1), and preeclampsia in the third trimester of pregnancy (n = 2). COVID-19 was detected in other patients during the hospital course. The duration of hospital admission was between 5 and 15 days for most of the patients. However, patient number 11 was hospitalized for 35 days, which is described in detail later. Four patients were admitted to the intensive care unit (ICU; patient numbers 6, 8, 10, and 11).

### Clinical characteristics

**Figure 1:**
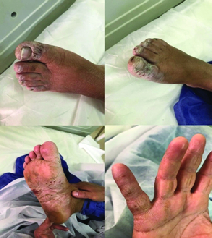
Cutaneous papulosquamous lesions with fissuring and erosions.

The most common clinical symptoms include shortness of breath (n = 10), dry cough (n = 10), myalgia (n = 8), and chills (n = 7). In addition, high-grade fever was found in 2 cases (temperature 41°C), 10 cases presented with low-grade fever (temperature < 38°C), and 5 cases were afebrile. Three cases had COVID-19-related papulosquamous lesions with fissuring that appeared before other manifestations of the disease (Figure 1). Moreover, decreased blood oxygen saturation (O2 saturation < 93%) was seen in four cases, and headache appeared in two patients. Two patients had a body mass index (BMI) of over 30 in this period of time (cases number 5 and 12). In terms of underlying diseases, six patients had hypothyroidism and were under treatment of levothyroxine with normal TSH, and one patient (case number 5) with ovarian dysgerminoma was under chemotherapy. Table I presents the patients' basic information and initial clinical characteristics.

### Paraclinical findings 

CXR reports showed pleural effusion in eight patients and ground glass appearance in five cases; however, three cases had normal CXR findings. High-resolution computed tomography (HRCT) added more information than X-ray to our findings in 11 patients. All details are provided in Table II.

Moreover, positive results of nasopharynx and oropharynx specimens for COVID-19 RT-PCR were reported in 12 patients. Leukopenia was not seen in our patients, except for case number 5, who developed leukopenia during the course of chemotherapy and was managed with one dose of granulocyte colony-stimulating factor (GCSF). However, leukocytosis (WBC 10400-20000 × 10${}^{9}$/L) was seen in eight patients. Elevated C-reactive protein (CRP) levels were seen in five patients. Thrombocytopenia was found in four patients without any other signs and symptoms of preeclampsia syndrome. Also, six patients had significantly increased liver enzymes (2-18 folds). Table II presents the laboratory results and imaging findings and differences between CXR and HRCT of all patients.

### Maternal and neonatal outcomes 

In total, we had one case of maternal mortality (case number 11), one of massive myocardial infarction, and one patient with pulmonary thromboembolism (PTE) with anticoagulant therapy. In cases number 8 and 10, hysterectomy was done due to placenta accreta. Among all cases, 12 pregnancies were terminated. Nine pregnancies were terminated with cesarean section (two cases in the second trimester admitted with premature labor pain (PLP) and terminated due to medication unresponsiveness and seven cases in the third trimester that were terminated due to preeclampsia [n = 2, PLP n = 2, and three cases due to term pregnancy]. Three In addition, three cases had normal vaginal delivery (two patients due to PLP and one case with term pregnancy). Among neonates, we had six cases of preterm labor (case number 1 was terminated due to PLP and HELLP syndrome at 29 wks of gestation; case number 4 had a vaginal delivery at home after being discharged from hospital in 26 wks of gestation due to PLP and premature rupture of membrane (PROM); cases number 6 and 11 was terminated due to PROM and PLP at 31 wks of gestation). We had 2 There were two cases of placenta accreta who were terminated at 21 and 24 weeks of gestation due to PLP and rupture of the uterus; both fetuses were expired. Also, we had one case of small for gestational age (SGA) who delivered at 36 weeks of gestation due to oxytocin challenge test positive result, one case of IUGR and mild preeclampsia at 37 wks of gestation, and one case of meconium staining at 39 wks of gestation. We had 8 Eight cases needed neonatal intensive care unit (NICU) admission after birth, as described in Table III. Eight neonates were breastfed according to health protocols; none of them had PCR positive test, illness, or death until now. Maternal and neonatal outcomes are summarized in Table III.

Medications such as hydroxycholoroquine, lopinavir, and oseltamivir were used for the management of COVID-19. Antibiotics such as meropenem, vancomycin, ceftriaxone, and azithromycin were also administered. All medications used in the course of hospitalization are listed (Table IV).

Here, we described the detailed hospital course of four patients with interesting clinical findings (cases number 4, 9, 10, and 11)

### Case number 4

A 24-yrs-old lady, Gravida 1, at 25 weeks of pregnancy, was admitted to hospital with chief complaint of headache, palpitation, and shortness of breath, and without any history of direct contact to COVID-19-infected patient. Her symptoms at the time of hospitalization included tachycardia (PR = 160/min), tachypnea (RR = 40/min), O${}_{2}$ saturation < 93% without using oxygen, and temperature 41°C. The patient did not have any sign of coughing, sneezing, or rhinorrhea but had palmoplantar papulosquamous lesions with fissuring with nailbed involvement. Her BMI was about 24 and did not have any past medical histories. Primary lab results showed lymphopenia (WBC 7200 × 10${}^{9}$/L, Lymphocyte 0.4%), mildly elevated liver enzymes (AST = 86, ALT = 56 U/L), CRP 96 mg/L, PT 15, and PTT 40.

There were no abnormalities in the primary CXR, but after two days of hospitalization, echocardiography was done for her due to persistent tachycardia, and bilateral moderate amount of pleural effusion was found incidentally. Additionally, HRCT was performed, and pleural effusion and the pleural band was detected in her left lung lower lobe. During the hospital course, the patient developed decreased platelet count (66000 × 10${}^{9}$/L, 38000, and 15000) with three episodes of epistaxis during five days. The result of the nasopharyngeal swab for COVID-19 RT-PCR was negative.

Based on these findings, antibiotic therapy and hydroxychloroquine were started for her, and the patient was admitted to the intensive unit care (ICU) ward. Also, lopinavir (200 mg/twice weekly) and IVIG (400 mg/kg/day) were started due to progressive thrombocytopenia and tachypnea. Urine protein test and LDH levels were in the normal range.

After five days of treatment, the platelet level returned to normal, and the patient's clinical symptoms improved gradually. After 14 days of hospital admission, she was discharged from the hospital. However, five days after her discharge, she developed a sudden onset rupture of membrane and labor pain and inevitably delivered a 900-gr baby at home. Her baby was admitted to the NICU ward and is currently in good condition there.

### Case number 9

A 37-year-old lady, Gravida 3 Live 2, at 37 weeks of gestation, was admitted to the hospital with the impression of IUGR and mild preeclampsia. She had a history of direct contact with a COVID-19-infected patient (her sister). At the time of hospitalization, the patient was febrile (38°C) and had a history of dry cough, anorexia, shortness of breath, myalgia, and abdominal pain since four days before the admission. Her past medical history was hypothyroidism and gestational diabetes mellitus (on medication) with a BMI of 28.8, and positive nasopharyngeal swab RT-PCR COVID-19 test.

Her pregnancy was terminated through the cesarean section route according to the gestational age. She was observed with the continuation of conservative management, hydroxychloroquine, and a prophylactic dose of anticoagulant. However, on the third postoperative day, the patient developed tachypnea and decreased oxygen saturation. A chest CT scan was performed for her, and the following findings were reported: bilateral pleural effusion and consolidation in the base of both lungs with a sign of clot formation in the right interlobar artery and posterior basal segment infiltration.

Heparin therapy was started for her with an impression of PTE. She was discharged after 14 days, with the administration of warfarin.

### Case number 10

A 34-year-old woman, Gravida 5 Abortion 3 Live 1 Dead 1 (with a history of two previous cesarean sections), was admitted to our hospital in a shock state at 24 weeks of pregnancy. She did not report any direct contact with a COVID-19 patient. On admission, she had decreased hemoglobin level (6 g/dl), and fast abdominopelvic sonography reported severe free fluid in her abdomen. She was immediately transferred to the operation room with the impression of placenta percreta and ruptured uterus in disseminated intravascular coagulation (DIC) state, and emergency hysterectomy was done. Subsequently, pelvis packing with long gauze was done due to massive transfusion and oozing from the site of operation. The patient was then transferred to the ICU ward after the operation. Her primary lab results included WBC 10,000 × 10${}^{9}$/L (Neutrophil count 7,400 and Lymphocyte count 2,000), platelet 36,000 × 10${}^{9}$/L, AST 560, ALT 170 U/L, PT 13, PTT 73, CRP 137 Mg/L, and ESR 26. Her nasopharyngeal sample for COVID-19 RT-PCR was negative; however, moderate pleural effusion of both lungs was reported in CXR. The abdominal pack was removed 48 hr after the operation, and the patient was extubated. Furthermore, bilateral pneumo catheter insertion was done, and pleural effusion was drained.

In the follow-up, HRCT results showed a pleural band in the lower lobe of the left lung with bilateral pleural effusions. She had negative results of pleural fluid culture and a normal level of procalcitonin. During the one week of hospital admission, the patient came out from DIC state and was transferred to the sub-ICU ward with good condition and normal echocardiography findings. Broad-spectrum antibiotics were administered during her course of hospitalization. On day 11 of admission, she developed cardiac arrest and posterior inferior ST-elevation myocardial infarction with troponin level over 2,000 ng/ml troponin. She was referred for emergency percutaneous coronary intervention (PCI). The result of PCI showed mild healed dissection in the mid and distal part of the main left coronary artery (MLCA). Echocardiography was done and reported 25% ejection fraction. Medical treatments were started for her. After 35 days of hospital admission and teamwork management, she was discharged in good condition, normal chest CT scan, and 35% ejection fraction in the last echocardiography report. Although her RT-PCR results were negative, her respiratory symptoms, cardiovascular complications, and primary lab results were in favor of COVID-19 infection.

### Case number 11

A 28-year-old lady, Gravida 5 Abortion 1 Dead 1 Live 2 (with a history of one previous cesarean section), was referred to our hospital with PLP and placenta accreta. The patient had past medical histories of hypothyroidism and minor thalassemia. On admission, she was febrile (temperature 38°C), and her first day of admission lab results included WBC 12,700 (Neutrophil count 6,850 and Lymphocyte count 4,950), platelet 138,000, and creatinine level 0.8 μmol/L. She was transferred to the operation room due to constant contraction and progression in vaginal examination patients. Cesarean section was performed with the impression of focal accreta. Six hours after the operation, the patient developed tachycardia (pulse rate = 140), tachypnea (respiratory rate = 24), and she was still febrile (38°C). Vaginal examination and abdominopelvic sonography were normal. Hemoglobin level was 11 × 10${}^{9}$/L, and electrocardiogram (ECG) showed sinus tachycardia. CXR was done and showed bilateral ground-glass appearance and bilateral pleural effusion.

The patient was transferred to the COVID-19 ward with the impression of COVID-19 pneumonia, and antibiotic therapy was started for her. She developed decreased urinary output (20 cc/hr) 24 hr later, her creatinine level increased to 1.3 μmol/L, and she still had tachycardia. Echocardiography was done that showed right-side heart failure and acute respiratory distress syndrome (ARDS) pattern with troponin level measuring about 2,000 ng/ml, and increasing AST level to 127U/L. She underwent conservative management. However, after 24 hr, she developed decreased O${}_{2}$ saturation < 93% and persistent tachycardia. PTE was ruled out for her according to spiral chest CT scan report. Echocardiography was performed again to find a cause for her tachycardia and O${}_{2}$ saturation decrease. She developed a cardiorespiratory arrest during the second echocardiography and died after 2 hr of resuscitation.

**Table 1:** Baseline information and clinical characteristics


**Patient number**	**Age (yr)**	**Gestational age (Wk)**	**Indication of admission**	**Fever${}^{a}$**	**Chills**	**Dry cough**	**Dyspnea**	**Myalgia**	**Skin lesions**	**Abdominal pain**	**Diarrhea**	**Decreased O${}_{2}$ saturation**	**Tachycardia**
**Patient 1**	25	29	PLP${}^{b}$	Low	_	+ +	_	_	_	_	_	_
**Patient 2**	39	15	COVID-19 pneumonia	High	+ +	+ _	+ +	+ _	_
**Patient 3**	19	39	COVID-19 pneumonia	Low	+ +	+ +	_	+ _	_	_
**Patient 4**	24	26	COVID-19 pneumonia	High	_	_	+ _	+ +	_	_	+
**Patient 5**	23	20	Ovarian dysgerminoma for chemotherapy	No	_	_	_	_	_	_	_	_	_
**Patient 6**	31	31	PLP	Low	+ +	+ +	_	_	_	+ +
**Patient 7**	34	36	Preeclampsia	No	_	+ _	_	_	_	_	_	_
**Patient 8**	40	21	PLP	No	_	_	_	_	_	_	_	_	_
**Patient 9**	37	37	Preeclampsia	Low	_	+ +	+ _	_	_	_	_
**Patient 10**	34	24	PLP	No	_	_	_	_	_	_	_	_	_
**Patient 11**	28	31	PLP	Low	_	_	_	_	_	_	_	+ +
**Patient 12**	32	39	COVID-19 pneumonia	No	+ _	_	+ _	_	_	_	_
**Patient 13**	32	10	COVID-19 pneumonia	Low	+ +	+ +	+ +	_	+ +
**Patient 14**	30	37	COVID-19 pneumonia	Low	_	+ +	+ _	+ _	+ +
**Patient 15**	24	39	COVID-19 pneumonia	Low	+ +	+ +	_	+ +	_	+
**Patient 16**	29	19	COVID-19 pneumonia	Low	+ +	+ +	+ _	_	_	_
${}^{a}$High-grade fever is defined as temperature > 38°C, ${}^{b}$PLP: Premature labor pain													

**Table 2:** Laboratory results, RT-PCR, and imaging findings of all patients


**Patient number**	**CXR**	**HRCT**	**WBC ×10${}^{9}$/L**	**PLT ×10${}^{9}$/L**	**CRP mg/L**	**AST U/L**	**ALT U/L**	**BUN mmol/L**	**Cr μmol/L**	**Pt**	**Ptt**	**U/P**	**PCR**
**Patient 1**	Bilateral pleural effusion	Bilateral pleural effusion lung consolidation	4200	23000	32	153	79	25	1.5	15.1	33	+2	+
**Patient 2**	Ground glass apearence	Ground glass apearence in both lung	4700	349000	96	17	13	6	0.7	13	30	- +
**Patient 3**	Normal	Normal	7000	202000	2.9	153	74	9	0.9	14	33	- +
**Patient 4**	Normal	Pleural band in lower lobe of left lung	5100	38000	96	86	56	10	0.6	15.3	40	- -
**Patient 5**	Ground glass apearence	Ground glass apearence in left lung	10400	166000	1	30	25	12	0.6	12	32	- +
**Patient 6**	Bilateral pleural effusion	Bilateral ground glass apearence, consolidation and pleural effusion	11300	221000	5	61	49	12	0.8	13	30	- -
**Patient 7**	Bilateral pleural effusion/ ground glass apearence	Bilateral consolidation and pleural effusion	20000	94000	77	29	22	12	1.4	13	33	+4	-
**Patient 8**	Bilateral pleural effusion	Increased pulmonary hypertension, bilateral minimal pleural effusion	5800	166000	120	24	10	12	0.4	12.6	28	+2	+
**Patient 9**	Bilateral pleural effusion	Bilateral consolidation and pleural effusion	6400	210000	30	32	21	6	0.5	13.1	28	- +
**Patient 10**	Bilateral pleural effussion	Pleural band in lower lobe of left lung + bilateral pleural effussion	10000	36000	25	560	170	13	1.8	73	33	- -
**Patient 11**	Ground glass apearence	Bilateral ground glass apearence, pleural effusion and pleural band	12700	138000	3	127	53	28	1.3	24	57	- -
**Patient 12**	Normal	Pleural effusion and pleural band	7800	105000	3	28	18	12	0.9	13	30	- +
**Patient 13**	Bilateral pleural effusion	Pleural effusion and pleural band	6300	217000	58	28	21	8	0.9	13.8	32	- +
**Patient 14**	Ground glass apearence	Bilateral ground glass apearence, pleural effusion	9600	393000	119	23	7	6	0.9	13	32	- +
**Patient 15**	Bilateral pleural effusion	Bilateral consolidation and pleural effusion	5800	171000	11	19	14	6	0.9	13	32	- +
**Patient 16**	Bilateral pleural effusion	Bilateral consolidation and pleural effusion	6600	183000	3	23	10	10	0.8	13	30	- +
CXR: Chest X-ray, HRCT: High-resolution computed tomography, WBC: White blood cell, PLT: Platelet, CRP: C-reactive protein, AST: Serum glutamic-oxaloacetic transaminase, ALT: Glutamic-pyruvic transaminase, BUN: Blood urea nitrogen, Cr: Creatinine, PT: Prothrombin time, PTT: Partial thromboplastin time, U/P: Urine protein, PCR: Polymerase chain reaction													

**Table 3:** Maternal and neonatal outcome


**Patient number**	**Need for ICU admission**	**Root of delivery**	**Prepartum complications**	**Intra-operation complications**	**Cause of termination**	**Post-operation complications**	**NICU admission**	**Breast feeding**	**Neonatal transmission**
**Patient 1**	_	NVD	_	_	PLP, HEELP	_	Yes/ Preterm	+ _
**Patient 2**	_	_	_	_	_	_	_	_	
**Patient 3**	_	C/S	_	_	SGA,OCT +	Abdominal wall hematoma	No	+ _
**Patient 4**	+ NVD	_	_	PLP, PROM	_	Yes/ Preterm	_	_
**Patient 5**	_	_	_	_	_	_	_	_	
**Patient 6**	+ C/S	_	_	PLP, PROM	_	Yes/ Preterm	+ _
**Patient 7**	_	C/S	_	_	Pre-eclampsia	_	Yes/SGA	+ _
**Patient 8**	+ C/S	_	Massive transfusion	PROM, Placenta Accreta	Pulmonary hypertension	No/dead	_	_
**Patient 9**	_	C/S	PTE	_	IUGR, Preeclampsia	PTE	Yes/IUGR	+ _
**Patient 10**	+ C/S	Rupture uterus	Massive transfusion	PLP, rupture uterus, placenta accreta	Myocardial infarction/ cardiomyopathy	No/dead	_	_
**Patient 11**	+ C/S	Maternal mortality	_	PLP	Right-sided heart failure/ ARDS	Yes/ Preterm	_	_
**Patient 12**	_	C/S	_	_	LP, BX	_	Yes/ Thick meconium	+ _
**Patient 13**	_	_	_	_	_	_	_	_	
**Patient 14**	+ C/S	_	_	LP	_	No	+ _
**Patient 15**	_	NVD	_	_	LP, ROM	_	No	+ _
**Patient 16**	_	_	_	_	_	_	_	_	
C/S: Cesarean section, NVD: Normal vaginal delivery, PTE: Pulmonary thromboembolism, LP: Labor pain, BX: Breech presentation, ROM: Rupture of membrane, ARDS: Acute respiratory distress syndrome, IUGR: Intra uterine growth retard, SGA: Small for gestational age													

**Table 4:** Medication used in the course of hospitalization


**Medications used**	**Hydroxy chloroquine 200 mg**	**Lopinavir 200 mg Q12 hr**	**Oseltamivir Q12 hr**	**Meropenem 1 gr Q8 hr**	**Vancomycin 1 gr Q8 hr**	**Ceftriaxone 1 gr Q12 hr**	**Azithromycin 250 mg Qd × 5 days**	**Heparin**	**Others**
**Patient No. 1**	+ 5 days		+ 14 days	+ 14 days		+ Mgso 4
**Patient No. 2**	+ 12days		+ 14 days	+ +	+
**Patient No. 3**	+ 14 days	+ 200 mg q12 hr	+ + 14 days		+ GSCF
**Patient No. 4**	+ 14 days	+ 200 mg q12 hr	+ + 14 days	+ +	+ IVIG
**Patient No. 5**	+ 14 days				+
**Patient No. 6**	+ 14 days		+ 14 days	+ +	+
**Patient No. 7**	+ 5 days		+ 14 days	+ +	+ Mgso 4
**Patient No. 8**	+ 5 days			+ +	Mgso 4
**Patient No. 9**	+ 14 days				+
**Patient No. 10**	+ 5 days	+ 200 mg q12 hr			+
**Patient No. 11**	_		+ 14 days	+	+
**Patient No. 12**	_				+
**Patient No. 13**	+ 5 days		+ 14 days		+
**Patient No. 14**	+ 5 days		+ 14 days		+
**Patient No. 15**	+ 5 days		+ 14 days		+
**Patient No. 16**	+ 5 days		+ 14 days		+

## 3. Discussion

The rapid spread of coronavirus disease needs timely intervention to protect the lives of mothers and fetuses infected with the virus. Despite the low risk of death with COVID-19 infection in the general population, we are concerned about the progression of the disease, the lower respiratory tract's involvement, and the need for invasive ventilation in this group of patients (17, 18). However, there are no reports of high-risk COVID-19 amongst pregnant women, and there is a lack of evidence that pregnancy increases the chance of getting the disease (19, 20).

The clinical appearances of SARS and MERS virus infection in pregnancy are different and include a wide range of clinical manifestations. The common clinical symptoms of COVID-19 patients include fever and dry cough. In our referral hospital, the most common symptoms of patients were low-grade fever, shortness of breath, and dry cough, while other symptoms like headache, abdominal pain, and diarrhea were less common (21, 22). Among the severe forms of the disease needing ICU care (cases number 4, 7, 8, 10, 11, 14), fever was detected in only 50% of patients upon hospitalization. Our findings are similar to Guan and colleagues (23). Therefore, it can be concluded that having a fever is not a specific criterion for determining the severity of the disease. Despite previous studies in which all patients were in the third trimester of pregnancy, we presented 1 case in the first, 5 in the second, and 10 in the third trimesters of pregnancy (12-16). For termination of pregnancies, the decision was made according to maternal-fetal conditions and gestational age (24).

On comparison of the findings of CXR and HRCT, it should be noted that in 12/16 cases, the chest CT scan added more information to CXR. CXR appeared normal in four cases (cases number 3, 4, 10, 12); however, in cases number 4 and 10, the presentation of disease and HRCT findings were different from CXR. The sensitivity of HRCT in the detection of COVID-19 pneumonia was about 98% higher than PCR (71%) (25).

HRCT was introduced as a noninvasive and conventional modality with high accuracy and sensitivity in detection of COVID-19 pneumonia in the early stages of the disease. Similar to Pan and colleagues (26), we could follow the progression of the disease or evaluate the response to medical therapy by using multiple chest CT scans. A chest CT scan can show the healing process even before the negative result of the RT-PCR test (26, 27). Although it cannot differentiate COVID-19 pneumonia from other viral types of pneumonia, we must consider COVID-19 diagnosis and isolate patients during this active pandemic period. Therefore, in suspicious patients with negative RT-PCR results, we considered a combination of exposure history, clinical symptom, and chest CT findings for detecting COVID-19 patients.

The most common finding in laboratory results was leukocytosis. The CRP level also had a direct contribution to the severity of the disease.

In critical patients with elevated liver enzymes and serum creatinine levels, a prerenal pattern was seen. Vascular emboli are associated with increased D-dimer and troponin levels, especially in the acute phase of the disease. Thrombocytopenia was seen as a severity sign of COVID-19 pneumonia in 40-45% of patients (28). We also had thrombocytopenia in critical cases that were related to multiple episodes of epistaxis.

Fang and colleagues*.* reported 71% sensitivity for the PCR test in the detection of the disease (25). Among the 16 patients admitted to our ward with suspicion of COVID-19 pneumonia, 11 had a positive nasooropharynx swab PCR test. However, among the six severe cases of the disease, only two had a positive nasopharyngeal PCR swab tests. According to recent studies, a positive PCR swab test is one of the most important criteria in the detection, isolation, and treatment of patients with COVID-19 (29). However, sometimes conversely, more positive PCR tests are seen in subacute cases rather than in critical cases with lower respiratory tract infection due to: immature development of nuclear acid detection technology; variation in the detection rate of the virus in different manufacture kits; lower viral load in the upper respiratory tract; improper clinical sampling and sampling during different periods of disease development and relatively long processing time. Therefore, the RT-PCR test is not highly sensitive for diagnosing lower respiratory tract infection with COVID-19 (25, 27).

We presented three critical cases with massive inferior-posterior wall myocardial infarction, PTE, acute right-sided heart failure, and death against prophylactic doses of heparin that was administered for them. COVID-19 can induce multiple organ injury with the following hypothetical mechanism: cytokine storm phenomena; hypoxic injury; coronary arteries spasm; and direct endothelial vascular injury that induces microthromboses (30). In case number 10, inferior-posterior wall STEMI occurred in the presence of nonobstructive disease on coronary angiography and led to myocardial injury with 25% cardiac ejection fraction in echocardiography. The troponin level was over 2,000 and 2,300 ug/ml D-Dimer level at 11 days of ICU ward hospitalization, respectively. Patients did not have any risk factors for heart disease. Also, abnormal coagulation parameter was reported in severe cases of COVID-19 pneumonia. It could lead to vascular thromboembolism events due to the dysregulation of urokinase pathways (31). In these cases, the PT and PTT time was prolonged.

The majority of women in our hospital affected by COVID-19 were treated with broad-spectrum antibiotics and hydroxychloroquine.

Hydroxychloroquine was used as a cost-effective, anti-inflammatory, and immunomodulatory drug to adequately penetrate the lung tissue. Because of these characteristics, it was considered as a treatment of COVID-19 pneumonia (32, 33). It is an experimental drug without any strong evidence of its efficacy for COVID-19 pneumonia. We used hydroxychloroquine in 12 cases (500 mg twice daily for 5-10 days). We also used lopinavir as an antiviral drug in two cases with severe form of COVID-19 who recovered (cases number 3, 4).

Several case-control studies of Covid 19 have examined the results of 41 pregnant women in the third trimester of pregnancy (12-16). The most common symptoms were fever and dry cough with increased CRP levels and liver enzymes. No severe illness was seen in any of them, and the infants' weight in these mothers did not differ from the normal population. Complications of pregnancy observed in them were similar to our observation, that is, PLP, PROM, and fetal distress. Viral PCR test did not detect virus particles in the amniotic fluid, umbilical blood.

Furthermore, throat swabs of neonates, breast milk, and maternal vaginal swabs did not detect viral particles. According to our findings, PLP and PROM were the most common complications that threatened COVID-19 mothers during the second and third trimesters. It can lead to catastrophic results, especially in mothers with a history of previous cesarean section (rupture of the uterus).

In SARS and MERS epidemic, vertical transmission in the perinatal period was not reported. In COVID-19, one infant with a positive PCR test just 30 hr after birth was reported as a questionable vertical transmission case. However, it has not yet been determined whether the disease was transmitted during pregnancy or after birth. Further, no vertical transmission in pregnancy on covid-19 was observed (12, 14). Viral PCR test of amniotic fluid, umbilical cord blood, throat swab of neonates, maternal vaginal swabs, and breast milk of mothers were negative in four recent studies (12-15). In this study, we had breastfeeding in eight cases without any reports of COVID-19 sign and symptoms in babies. Although there is not enough data to determine how long the infected mother should separate from the baby, so we advised mothers to follow the health tips techniques during breastfeeding until complete recovery.

Based on the available findings to reduce maternal and neonatal complications during the COVID-19 pandemic, the following suggestions can be given in the diagnosis and treatment of pregnant women: (i) Early diagnosis of COVID-19 can be made using the combination of exposure history, clinical symptoms, chest CT findings, and RT-PCR test for detecting Covid19 women. (ii) Also, it is important to pay attention to certain lab results in the severe form of the disease, including thrombocytopenia, elevated D-dimer, liver enzyme (AST, ALT) levels, and their course of progression of the disease. (iii) Team-based approach and multispecialty consultations are needed in the management of these patients. (iv) Empiric antibiotic therapy should be administered to prevent the second bacterial infection risk in these patients. (v) Fetal and uterine contraction monitoring should be done in these patients. (vi) Individual delivery plan should be given to pregnant COVID-19 patients based on their condition.

## 4. Conclusion

Clinical manifestations and paraclinical results of pregnant women with COVID-19 were similar to non-pregnant COVID-19 patients. Fever was detected in only 50% of the patients upon hospitalization; therefore, it is not a specific feature for the diagnosis of the COVID-19 during pregnancy. Cutaneous lesions can be the first symptoms in COVID-19 pregnant patients. There is no evidence of vertical transmission in our cases. PLP and PROM were the most common complications in the second and third trimesters of pregnant COVID-19 women, which can lead to rupture of uterus in those with a history of previous cesarean section. Termination and delivery plans should be given individually based on each patients' condition.

##  Conflict of Interest

There are no conflicts of interest for the authors to report.
